# Structured work-based learning in undergraduate clinical radiology immersion experience

**DOI:** 10.1186/s12909-021-02592-0

**Published:** 2021-03-17

**Authors:** Ulf Teichgräber, Maja Ingwersen, Florian Bürckenmeyer, Amer Malouhi, Clemens Arndt, Aimée Herzog, Tobias Franiel, Hans-Joachim Mentzel, René Aschenbach

**Affiliations:** Department of Diagnostic and Interventional Radiology, Friedrich-Schiller-University Jena, Jena University Hospital, Am Klinikum 1, 07747 Jena, Germany

**Keywords:** Distance education and online learning, Immersion learning, Undergraduate medical education, Peyton’s four-step approach, Workplace learning

## Abstract

**Background:**

Practical courses in undergraduate medical training often lack a didactic concept. Active participation and learning success largely depend on chance. This study was initiated to evaluate a novel concept of structured work-based learning (WBL) in the course of students’ half-day radiology immersion experience (IE).

**Methods:**

This prospective, single-centre cohort study included 228 third-year students of the 2019 summer semester who underwent the obligatory radiology IE at a university hospital. The course was based on a novel structured WBL concept that applied established didactic concepts including blended learning, the FAIR principles of feedback, activity, individualization, and relevance, and Peyton’s four-step approach. Outcomes of equal weight were student and supervisor satisfaction with the clinical radiology IE assessed by paper-based- and online survey, respectively. Secondary outcome was achievement of intended learning outcomes assessed by means of mini clinical evaluation exercises and personal interviews.

**Results:**

Satisfaction with structured WBL was high in 99.0% of students. Students’ expectations were exceeded, and they felt taken seriously at the professional level. Dissatisfaction was reasoned with quality of learning videos (0.6%), little support by supervisors (0.5%), or inadequate feedback (0.6%). Supervising resident physicians rated achievement of intended learning outcomes regarding cognitive and psychomotor competences as excellent for all students. Personal interviews revealed achievement of affective competence in some students. Twelve of 16 (75.0%) supervising physicians were satisfied with focussing on intended learning outcomes and student preparation for IE. Two of 15 (13.3%) supervisors were unsatisfied with time spent, and 4 of 16 (25%) with the approach of assessment.

**Conclusions:**

This study demonstrated that both students and supervisors were satisfied with the novel concept of structured WBL within the scope of clinical radiology IE. Achievement of intended learning outcomes was promising.

**Supplementary Information:**

The online version contains supplementary material available at 10.1186/s12909-021-02592-0.

## Background

Work-based learning (WBL) in general refers to learning in a work setting as part of a formal education program. In Germany, work-based learning regarding clinical radiology is offered to third-year medical students as mandatory half-day clinical radiology immersion experience (IE) and, as a matter choice, as 3-month part of the sub-internship. During this time, medical students should apply their theoretical knowledge and practical skills in real workplaces at the radiology department.

Although, WBL is considered as basically useful, lack of student and supervisor motivation could jeopardize success [1]. In our department of radiology, students complained that during their clinical radiology IE, they primarily had to watch resident physicians’ professional activities. They passively had to shadow their supervisors throughout the workday. Students’ IE had neither been structured in terms of learning objectives and time, nor in support. No specific didactical concept was applied. Consequently, students’ satisfaction was low and strongly depended on the individual commitment of the supervising radiologist. On the other hand, supervisors reported that students had not been prepared well for IE and supervision had increased their workload to the extent that students may not benefit from radiological IE appropriately.

To break this vicious circle and to increase both motivation and success, we developed and implemented a novel WBL concept for the half-day radiological IE that provided students with structured opportunities of supervised activities (e.g.: interview patients, inform patients, reading images, writing reports). Responsibilities between all participants were arranged within clear timelines. The structure of WBL was based on skill-centred intended learning outcomes (ILO) [2] to be achieved in compliance with the established didactic concepts of blended learning [3, 4], FAIR principles [5], and Peyton’s four-step approach [6]. We incorporated blended learning in form of learning videos for self-determined preparation. The FAIR principles had to be respected by providing feedback, facilitating students’ professional activities, assuring individualization by selectable workplaces, and communicating relevance of ILOs. We used Peyton’s four-step approach to convey occupationally specific skills and to achieve a lasting learning success. The purpose of our study was to evaluate satisfaction with structured WBL in the course of the half-day clinical radiology IE among both students and supervisors.

## Methods

### Study design

Our prospective, single-centre cohort study included third-year medical students of the summer semester 2019 who underwent the obligatory half-day clinical radiology IE at the department of radiology of a German university hospital. We designed our pre-experimental study for post-test only evaluation (students) and pre-test-post-test evaluation (physicians), respectively [7]. The radiology IE takes place in the first year of students’ clinical part of medical studies after termination of the preclinical part. In parallel, in this period, students attend their first lectures on clinical radiology. Senior- and resident physicians participated in the study as supervisors according to their regular duty rosters. Students participated in the newly implemented concept of structured WBL that was based on three established didactic approaches. First, we applied blended learning [3, 4] by providing students with learning videos to prepare themselves for topics they had to pass through during the IE. Second, we incorporated the FAIR principles (feedback, activity, individualization, and relevance) [5]. And third, we implemented Peyton’s four-step approach (demonstration, deconstruction, comprehension, and execution) [6].

Medical students self-determinedly prepared for the practical radiology IE by means of two learning videos (10 min each) on the obligatory topics of the practical course. During the four-hour IE at the department of radiology, two students were allocated to one responsible senior physician and one student to one resident physician at each workplace. The actual practical training started with a 30-min guided tour around the department of radiology conducted by the responsible senior physician. Subsequently, students started their work at the first workplace. Here, supported by a resident physician, they had to read chest X-ray images and to create a medical report. At the second workplace, students had to inform a patient on preparation for magnetic resonance imaging (MRI) or computed tomography (CT) including the administration of contrast agent, and to obtain the patient’s written informed consent (under the permanent control and under the responsibility of the supervising physician). Resident physicians had to assess student’s procedural skills at both obligatory workplaces separately by means of mini clinical evaluation exercises (mini-CEX) (Supplemental information: Additional file 2). Residents were encouraged to directly explain and discuss fulfilled and unfulfilled requirements with the students (optional feedback). Afterwards, students attended the third, elective workplace. Overall, they spent 1 h at each single workplace.

**Fig. 1:**
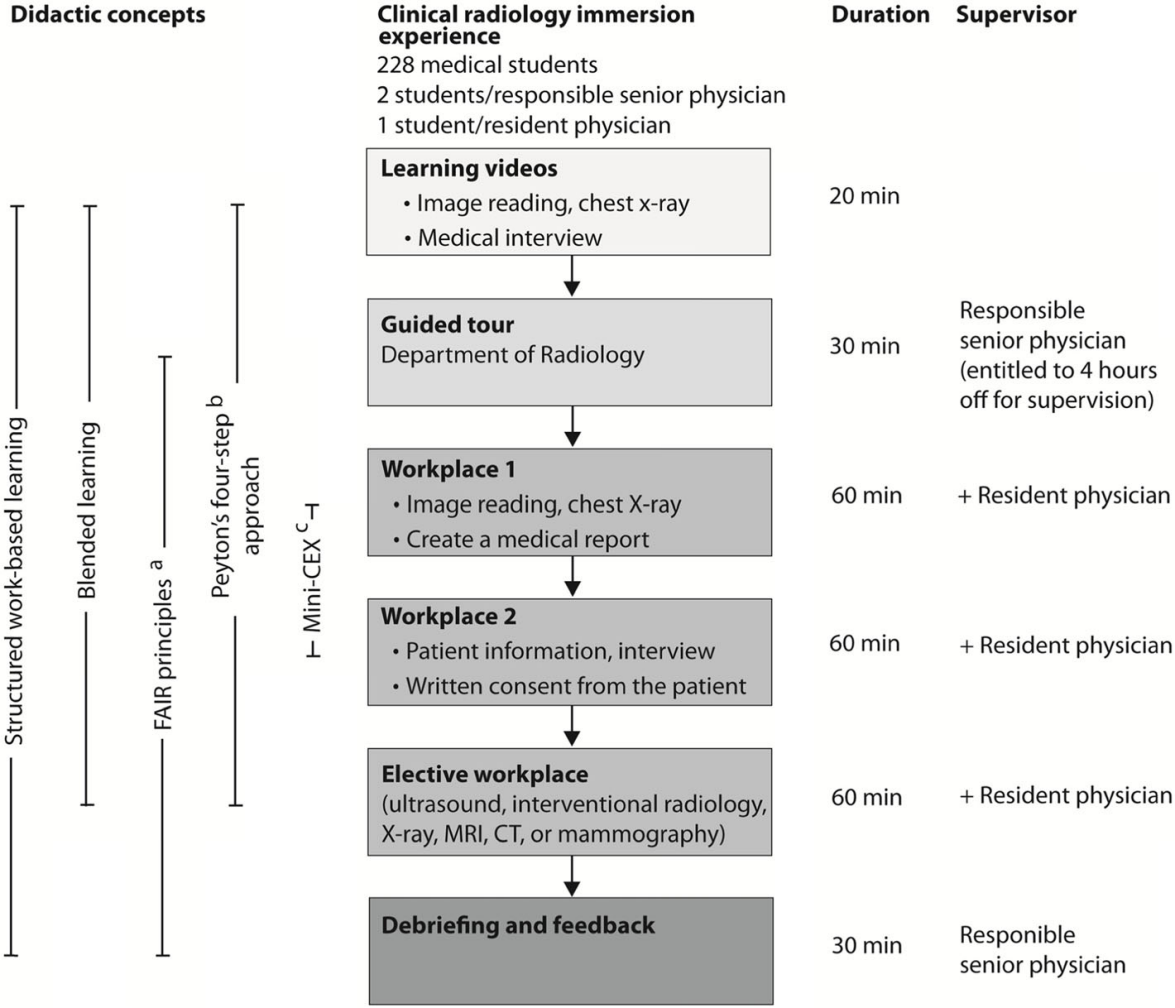
Flowchart of the clinical radiology immersion experience of a study on work-based learning. ^a^ FAIR principles: feedback, activity, individualization, relevance. ^b^ Peyton’s four-step approach: demonstration, deconstruction, comprehension, execution. ^c^ Mini-CEX: mini-clinical evaluation exercise

Following the proceedings through the workplaces and equipped with their medical report, patient’s written informed consent, and the two completed mini-CEX forms, students underwent a 30-min debriefing including final feedback on their performance concerning the ILOs. Debriefing and feedback were conducted by the responsible senior physician (Fig. 1).

Immediately after the feedback, we asked all students to participate in a paper-based survey on seven issues regarding their satisfaction with the new concept of the structured WBL experience. In addition, research assistants interviewed volunteer students on their experience. We invited all supervisors to participate in an online survey (Lime survey: an open-source survey tool; LimeSurvey GmbH, Hamburg, Germany). Questions corresponded to three- or five-point bipolar Likert scales. For evaluation, we anonymized all survey data, and thus, local ethics committee exempted our study from the obligation of approval.

### Intended learning outcomes

Before the practical course took place, we described and communicated ILOs according to Biggs and Tang [2] to the students:

1. I am able to apply a systematic approach to read a chest X-ray image and to prepare a radiological report.
2. I am able to conduct patient information on preparation for MRI/CT.
3. I am able to reflect relevance of acquired knowledge and skills in clinical radiology.

ILOs were chosen due to relevancy for students’ later professional practice. Description was student-centred and thus, emphasized the value the radiology IE should bring to the students (principle of “What’s in it for me?” [WIIFM]) [8]. During IE, medical students should have been engaged to activities most appropriate to the ILOs. Already in the planning phase of the structured WBL concept, we aimed to establish constructive alignment [2, 9] across ILOs, student’s activities at workplaces, and assessment of tasks. We aligned ILOs to real-world clinical situations.

### Applied didactical concepts

In the course of the radiology IE, we mainly based structured WBL on the application of three didactical approaches: blended learning, FAIR principles, and the Peyton’s four-step approach (Fig. 1).

### Blended learning

Blended learning [3, 4, 10, 11] in our study designates the combination of a pre-IE online delivery of content and an instruction that combines learning videos with face-to-face experience at workplaces. Beforehand, the radiology faculty had prepared two experience-level appropriate videos on both obligatory workplace topics of 10 min in length each. Videos were available on the university content management system for students at all times and students were asked to watch them for clinical radiology IE preparation. Students were already familiar with the concept of blended learning because it is routinely applied in other radiology lectures, seminars, and courses.

### FAIR principles

Harden et al. [5] had introduced the FAIR principles of teaching to increase effectiveness of learning. Principles include the recommendation to provide appropriate feedback to students, to engage active learning, to align learning to individual needs, and to make learning relevant to intended outcomes. We incorporated the four principles into the structured WBL concept (Supplementary information: Additional file 1).

1. During their radiology IE, students received optional timely feedback from the resident physicians in the course of two mini-CEX on the obligatory workplace topics. In addition, at the end of radiology IE, the responsible supervisor provided a closing feedback. We instructed supervisors to be explanatory and specific. Feedback should refer to ILOs.
2. Active engagement assures sustainable learning success. Thus, students were actively involved in the diagnostic clinical setting of the workplaces at the department of radiology.
3. Students could choose the radiologic topic of their third workplace that best suited their individual needs. Six options were available: ultrasound examination, interventional radiology, X-ray, magnetic resonance imaging, computed tomography, and mammography.
4. We applied relevance as criterion for selection of the workplace topics and the corresponding assessment tasks. In the clinical context, students can realize usefulness of ILOs by applying theory into practice.

### Peyton’s four-step approach

We integrated the task-centred Peyton’s four-step approach [6] into the structured WBL concept. The first step of demonstration included watching of learning videos and observing and listening to the resident physician at the workplace.

During the second step of deconstruction, the resident slowly repeated the respective activity and described each procedural step in detail. Students may have asked questions. The third step of comprehension allowed the student to guide the resident through the procedure. Finally, the fourth step of execution consisted of independent performance of the activity by the student on his/her own (Table 1).

**Table 1:** Peyton’s Four-Step Approach for the Work-Based Learning Immersion Experience in Clinical Radiology

Peyton’s four steps to acquiring skills	Chest X-ray image reading	Patient information
**Demonstration**	• Pre-IE learning video • At workplace 1, resident physician read X-ray image and dictated a radiological report at normal speed. • Student listened and observed.	• Pre-IE learning video • At workplace 2, resident physician conducted patient information and obtained written informed consent on MRT/CT. • Student listened and observed.
**Deconstruction** “Talk the trainee through”	• Resident physician explained the reporting rationale and algorithm slowly, step by step while dictating the report. • Student listened and asked questions.	• Resident physician explained rationale and algorithm of patient information slowly, step by step while conducting the interview. • Student listened and asked questions.
**Comprehension** “Trainee talks the trainer through”	• Student explained every single step of the reporting algorithm before resident put it into practice. • Resident physician dictated the radiological report.	• Student explained every single step of patient information before resident put it into practice. • Resident physician conducted the patient information.
**Execution** “Trainee does”	• Student read X-ray image and dictated the report. • Resident supervised.	• Student informed patient on preparation for MRT/CT and obtained written informed consent. • Resident supervised.

### Outcomes

Primary outcomes of equal weight were student and supervisor satisfaction with the clinical radiology IE according to paper-based and online survey, respectively. Secondary outcome was achievement of ILO by mini-CEX and personal interview.

### Assessment of intended learning outcomes

Residents assessed clinical performance of students regarding the first two ILOs at each obligatory workplace using the tool of mini-CEX [12]. They based their assessment on direct observation of the student skills in the clinical situation. Radiographic reports were assessed based on clinical impression, structure, style, form, and wording. Mini-CEX rating forms consisted of 11 domains on the first ILO (workplace 1) and 7 domains on the second ILO (workplace 2) (Supplementary information: Additional file 2). Domains of mini-CEX included cognitive and psychomotor competencies [7]. Cognitive domains covered factual knowledge (e.g.: naming of pathologies), problem solving (e.g.: identification of pathologies, organisation), and clinical decision making (e.g.: create a medical report). Psychomotor competencies included behavioural competencies (e.g.: checking patient data) and skill competencies (e.g.: visual: checking image quality; hearing: medical interviewing; speech: patient education). Residents rated each particular competence on a three-point scale (worthy of improvement, requirements met, excellent performance). Mini-CEX included an optional small oral and written feedback on student’s strengths and suggestions for improvement. Students handed completed mini-CEX forms to the responsible senior physician at debriefing to be used in an effort to provide a final formative and summative feedback [10, 13]. The third predefined ILO that covers affective learning (whether students value the importance of learning) will be assessed in personal interviews by research assistants.

### Statistical analysis

Categorical data are presented as counts and percentages. Differences between senior and resident physicians were assessed with Fisher’s exact test. *P*-values refer to comparison of proportions of satisfied and unsatisfied votes between senior and resident physicians without consideration of neutral votes. A 2-sided value of *p* < 0.05 was considered as statistically significant. We performed analysis using XLSTAT (Version 2015.6.01.24026, Addinsoft, Paris, France).

## Results

### Participants

In the summer semester 2019, we prospectively enrolled a total of 228 undergraduate medical students into our single centre observational experience on structured work-based learning related to the clinical radiology IE at the department of radiology of a German university hospital. A total of 85.5% students (195 of 228) completed the paper-based student survey and personal interview, and 61.5% of physicians (16 of 26) completed the online supervisor survey (senior physicians 71.4% [10 of 14], resident physicians 50% [6 of 12]). Resident physicians rated mini-CEX of all students as “excellent performance” in all domains.

### Student survey

**Fig. 2:**
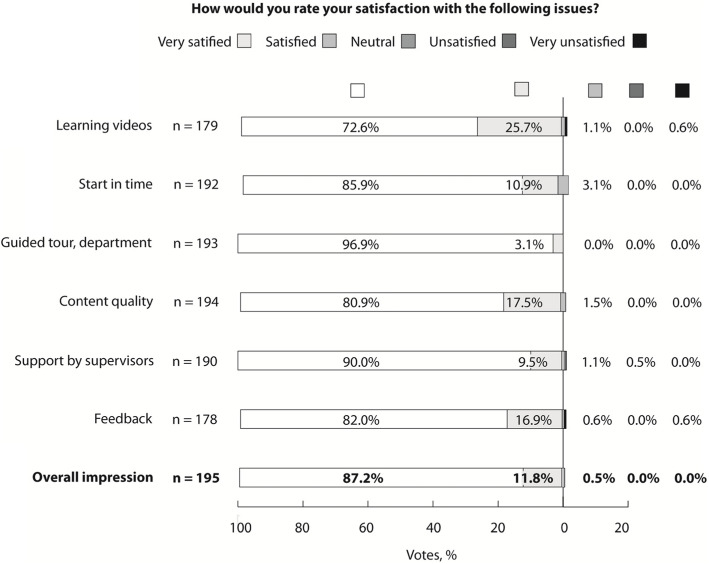
Medical student survey on satisfaction with the concept of structured work-based learning in the course of the clinical radiology immersion experience. Totals may be subject to discrepancies due to rounding

In general, the majority of students were very satisfied (87.2%) or satisfied (11.8%) with the structured work-based learning they experienced during clinical radiology IE. The proportion of unsatisfied students was low (0.5% regarding support by supervisor and 0.6% regarding learning videos and feedback, respectively) (Fig. 2).

Personal interviews immediately following the radiological IE revealed that preparatory learning videos were considered useful and attractive. Self-determined preparation increased students’ confidence at the workplaces. However, students criticised a partially poor transposition such as lengthy reports on too many details or insufficient video sound quality. They suggested to provide handouts and additional videos on elective workplaces.

Regarding the obligatory topics of chest X-ray and patient information, students were divided according to appropriateness. Some of the students would have preferred radiology-specific topics such as MRI and CT instead of patient information.

Support from supervisors was deemed best in chest X-ray image reading. Due to the high workload at the CT workplace at the emergency department, some of the students experienced poor support at their elective workplace. Under conditions of visual and acoustic unease, some students would have preferred a separate room for patient information. Closing feedback was generally experienced as appreciative. Students felt themselves to be taken seriously by supervisors. However, in the view of a few students, feedback was redundant.

Timetable and switching between workplaces were broadly accepted. In addition, students suggested to take greater account of the patient’s way through the diagnostic steps to gain overview of the radiological pathway. Finally, students were positively impressed by the organisation of the radiological IE. Their expectations were exceeded. IE not only gave insights and increased occupational identity but also got some of the participants strongly interested in the field of radiology (Table 2).

**Table 2:** Debriefing of 195 Undergraduate Medical Students on the Clinical Radiology Immersion Experience Under Application of Structured Work-Based Learning

Interview question	Positive aspects	Negative aspects	Suggestions for improvement
**Videos** How would you assess the WBL training with regard to learning videos?	• Videos were a good preparation for IE. • Contents were well communicated and effectively imparted. • Videos could be viewed repeatedly. • Videos for preparation should be considered as standard. • Videos increased self-confidence. • Videos increased attention.	• Video on patient information: too many details, long-winded. • Sound quality was unsatisfactory.	• Handouts for learning videos could be useful • Patient informed consent forms should be provided. • Additional videos on elective workplaces could be desirable.
**Topics** In your estimation, where the choice of radiological topics appropriate for WBL?	• Very good selection of topics.	• Would have preferred magnetic resonance imaging (MRI) over patient information. Patient information is also taught at other departments. • Would have preferred MRI or CT over patient information.	• Handouts alone might be sufficient for the topic of patient information.
**Interview question**	**Positive aspects**	**Negative aspects**	**Suggestions for improvement**
**Support** When did you feel most comfortable? When did you miss support?	• Supervisors were strongly committed. • Initial uncertainty was quickly overcome. • Feedback made students feel being taken seriously by supervisors. • Immediate feedback on student’s own performance. • Particularly good support for imaging reading.	• Support at the central emergency room computed tomography depended on the workload at the department.	• A separate room for patient information could be provided.
**Program** How would you assess the workflow?	• IE was well structured. • Four-hour IE was appropriate to sustain concentration and receptivity. • Switching between workplaces preserved suspense for students.	• Too little time. • Feedback was unnecessary.	• Would be happy to participate in the early meeting. • It would be helpful to follow the complete path of the patient to gain overview of the different steps. • Wish to spend more time at the department of radiology. But no longer than 4 h per day. • Wish to extend IE up to 6 h.
**Interview question**	**Positive aspects**	**Negative aspects**	**Suggestions for improvement**
**Expectations** Did the radiology WBL-based IE meet your expectations?	• IE turned out to be better than expected, in particular regarding organization and feedback. • Positively impressed • Never experienced like this before.		
**Significance of clinical radiology** How do you assess the importance of radiology for clinical diagnostic?	• Relevance of radiology became increasingly clear.		

### Supervisor survey

**Fig. 3:**
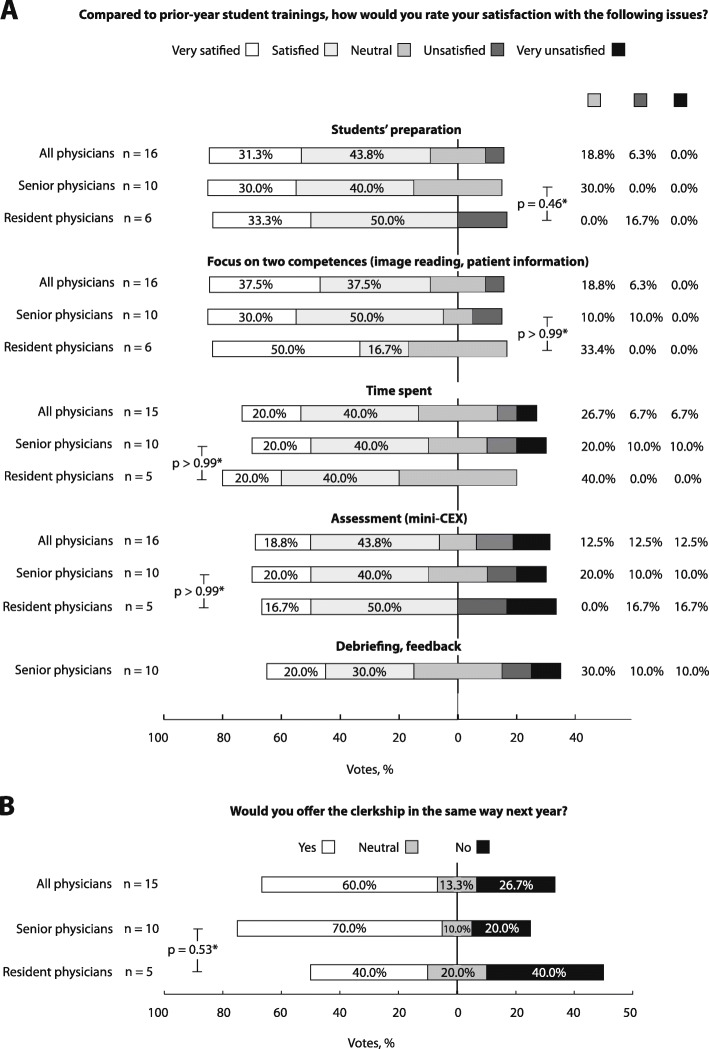
Supervisor online survey regarding satisfaction with the concept of structured work-based learning in the course of the clinical radiology IE (**a**) and vision for the future (**b**). Totals may be subject to discrepancies due to rounding. * *p*-values refer to comparison of proportions of satisfied and unsatisfied votes between senior and resident physicians without consideration of neutral votes

A total of 75% supervisors (12 of 16) were satisfied with preparation of students for the workplaces by means of learning videos. However, they stated that some of the students had admitted not to have watched the videos and thus, numbers might be underreported. Regarding obligatory topics, 75% of supervisors were satisfied. One senior physician complained on the limited individual freedom to teach. Two senior physicians (13.3%) were unsatisfied with the time necessary for supervision and two senior and resident physicians respectively (25%) were unsatisfied with the mini-CEX evaluation questionnaire. The rating of student preparation, obligatory topics, time spent, and mini-CEX did not differ significantly between senior and resident physicians. Fifty percent (5 of 10) of senior physicians rated the value of final feedback as high and 20% (2 of 10) as low (Fig. 3a).

About two thirds (60% [9 of 15]) of the supervising physicians would offer a clinical radiology IE in accordance with the underlying concept of work-based learning once again (Fig. 3b). They acknowledged the clear guidance and the improved quality of structure and organisation.

## Discussion

Our single centre, observational study evaluated satisfaction with structured WBL among students and supervisors in the course of a four-hour clinical radiology IE. Core of structured WBL was constructive alignment [2] of competence- and skill-centred ILOs with content of preparatory learning videos, workplace activities, assessment, and feedback. The study demonstrated that the majority of students were satisfied with the structured manner of IE. They felt professionally appreciated. A few shortcomings regarding technical and didactic quality of learning videos and temporarily limited support by supervisors in case of high workload at the emergency department were reported. Satisfaction of supervisors was somewhat lower, in particular regarding assessment of ILOs by mini-CEX and time spent for supervision.

At workplaces, students were supported and challenged by resident physicians to participate and apply their competences. Applying clinical knowledge and skills with actual patients, requires students to organize and recall information and to use heuristics (“mental shortcuts”) representing a high level of academic performance [14]. Thus, we expected our students to achieve high level ILOs. In accordance with Biggs and Tang [2] we assume that motivation and learning is enhanced not only with relevance but also with challenging ILOs. Timely communication of ILOs and constructive alignment to all steps of the radiology IE up to the final feedback were the backbone of structured WBL. In the future, a team of supervisors might be involved to a greater extend in the decision and revision process on ILOs.

Previous studies found learning videos to be as effective as live lectures in preparation of medical exams [15]. Afzal and Masroor [16] reported on no difference in outcomes of the end-of-radiology clerkship test regarding chest X-ray interpretation between students who underwent blended learning or traditional lecture. Videos allow students to determine processes and to repeat the lecture at any time. In addition, preparation gains time and encourages student confidence at workplaces. Thus, in our opinion, self-determined learning by watching videos on ILOs served as appropriate preparation for workplace activities. However, “demonstration” is only the first step of Peyton’s approach and further steps including active decision making and performance are required for lasting success.

Self-determined pre-IE preparation and the one-to-one assignment between student and resident supervisor may have helped to increase students’ confidence and communication at workplaces, and, in turn, to improve motivation and performance of ILOs [17]. Throughout the IE, students were supported to change from observing into contributing participation. They had to step outside their comfort zone.

Peyton’s four step approach that progressively leads students to clinical performance, had already been proven to be superior over standard instruction [18] with Peyton’s step 3 (“trainee talks trainer through”) accounting for the biggest share of success regarding task and memory performance compared to steps 1 and 2 [19]. Observation of skills combined with (motor) imagery of performance is considered to be superior to observation alone.

Support to students was provided in form of organizational conditions of radiology IE structure including faculty resources, timetable, and organized activities, in form of formal didactic support by supervisors, and by affective support through positive attitudes towards students [20]. In addition, due to increased consideration of resources and time management, structured WBL might have given supervising residents greater backing for the challenge to manage student training, patient needs, and administrative duties.

Key objective of the assessment of clinical skills is to facilitate learning [21]. The assessment tool of mini-CEX was aligned with ILOs, activities at the workplaces, and the feedback. Assessment intended to cover how well students achieved the ILOs, and not how well they reported what supervisors told them. However, resident physicians, who conducted the formative assessment were potentially biased by the relationship with their students and their process of learning at the workplace. As a result, in our study, residents consistently judged students’ performance as excellent. Poor accuracy of faculty assessment was already described previously [22, 23]. Senior physicians had to provide a more objective debriefing with the help of active reflection by students. In some cases, this approach resulted in duplications and contradictions. In the future, mini-CEX might serve more as a checklist of specific observed tasks rather than a rating tool and emphasize residents’ suggestions of improvement. In general, the major purpose of feedback is to reduce the discrepancy between current and desired practices or competences [24]. Thus, final debriefing with senior physician may include a responsive feedback dialog [25] to provide a “plan of action” and advice students regarding available resources [13].

Our study has some limitations. First, it was a single group, pre-experimental evaluation to document proficiency and process of structured WBL in clinical radiology IE. We did not compare participants’ satisfaction or outcomes of structured WBL with the traditional, radiological IE. Therefore, accomplishments may be due to factors other than the novel structured WBL concept [7]. In addition, mini-CEX did not reflect actual competence of students due to probable rater biases and thus, did not provide reliable assessment of achieved ILOs, but rather encouraged students on their path towards professional competence. Therefore, at that time, we cannot quantify efficacy of structured WBL.

## Conclusions

Structured WBL in the course of the clinical radiology IE was feasible and well accepted among participating students and supervisors. Students felt appreciated at the professional level and supervisors were supported by organizational conditions. From our study, we derived to continue offering structured WBL at the department of radiology. However, both diversity and quality of learning videos should be pursued. In addition, assessment and feedback should place particular emphasis on providing students with information where and how to improve or broaden their competences rather than to judge their abilities. Efficacy of structured WBL regarding learning success and sustainability has still to be demonstrated.

## Supplementary Information

**Additional file 1.**

**Additional file 2.**

## Data Availability

The datasets generated and analysed during the current study are not publicly available due to requirements of the ethics committee but are available from the corresponding author on reasonable request.

## Abbreviations

FAIR principles: Principles off feedback, activity, individualization, and relevance
ILO: Intended learning outcomes
Mini-CEX: Mini clinical evaluation exercises
WBL: Work-based learning
