# Supplementary material for: Structured work-based learning in undergraduate clinical radiology immersion experience
Source: BMC Med Educ. 2021 Mar 17;21:167. doi: 10.1186/s12909-021-02592-0 (PMC7972199; doi:10.1186/s12909-021-02592-0)
Supplement: Supplementary file 1 — Additional file 1. [file 12909_2021_2592_MOESM1_ESM.docx]

**Structured Work-Based Learning in Undergraduate Clinical Radiology Immersion Experience**

**Supplementary information**

**Additional file 1**

| **Table** FAIR Principles of Teaching and Learning in Medicine according to Harden et al. (2013) [1] | | |
| --- | --- | --- |
| **F** | Provide feedback | - Give students an explanation of the assessment to enable them to improve their performance. - Provide specific feedback about performance against predefined intended learning outcomes. - Provide timely feedback. - Be adequately prepared for feedback. - Help students to plan their further learning. |
| **A** | Engage the student in active learning | - Ensure that students be encouraged to be actively involved in patient care activities. |
| **I** | Individualise learning | - Provide a range of learning opportunities. - Up to a third of curriculum time may be allocated for electives. |
| **R** | Make the learning relevant | - Applying theory in praxis motivates to learn. |

[1] Harden RM, Laidlaw JM. Be FAIR to students: four principles that lead to more effective learning. Med Teach 2013; 35(1):27-31.
