# Supplementary material for: Structured work-based learning in undergraduate clinical radiology immersion experience
Source: BMC Med Educ. 2021 Mar 17;21:167. doi: 10.1186/s12909-021-02592-0 (PMC7972199; doi:10.1186/s12909-021-02592-0)
Supplement: Supplementary file 2 — Additional file 2. [file 12909_2021_2592_MOESM2_ESM.docx]

**Structured Work-Based Learning in Undergraduate Clinical Radiology Immersion Experience**

**Supplementary information**

**Additional file 2**

**Do not** hand over the form to the student(s) before mini-CEX.

The completed sheet will be handed in by the student to the senior physician for discussion and will remain at the radiology department.

**Mini-Clinical Evaluation Exercise - Reporting X-Ray Thorax**

**General-RAD Paediatric-RAD Neuro-RAD**

**Name: _________________**

**Semester: _________________**

**Date _________________**

| **Competence** | Worthy of improvement | Requirements met | Excellent performance |
| --- | --- | --- | --- |
| Verification of patient data and questions | **** | **** | **** |
| Checking image quality | **** | **** | **** |
| Recognition and naming of external material | **** | **** | **** |
| Recognition of a pneumothorax | **** | **** | **** |
| Description of the configuration of the mediastinal structures | **** | **** | **** |
| Determination of the heart-thorax quotient | **** | **** | **** |
| Identification and naming of lung pathologies | **** | **** | **** |
| Recognition and description of pleural effusions | **** | **** | **** |
| Identification and naming of pathologies of the bony thorax and soft tissue mantle | **** | **** | **** |
| Creating a medical report | **** | **** | **** |
| Organisation/Effectiveness | **** | **** | **** |

**Today the student has the requirements for the above-mentioned skills...**

... not fulfilled (= **significant deficiencies** regarding a technically correct, complete, and flowing course)

... fulfilled (= **at least sufficiently technically correct**, complete and fluent to pass)

... excellently fulfilled (= excellent, **far above-average** performance)

Optional feedback to the student(s):

Strengths:

Suggestions for improvement:

Name of the examining physician:

Signature:

**Implementation of the Mini-CEX**

In order to optimize image reading, the intended learning outcome should be taught to the students in the following fixed Peyton scheme:

| 1. Demonstration | The assistant doctor reads an image and generates the radiological report in normal/routine speed. The student observes. |
| --- | --- |
| 1. Deconstruction | The assistant doctor generates the report slowly and explains each of the steps. The student may ask if necessary. |
| 1. Understanding | According to the student's instructions, the assistant doctor generates the report. If necessary, student’s instructions will be corrected and supplemented. |
| 1. Performance | The student independently generates a radiological report |

The radiological report is then evaluated by the assistant physician with the mini-CEX sheet. Incomplete or insufficient competencies are then discussed. If a debriefing is necessary for a large number of competences, i.e. if there are considerable deficiencies, the section of the daily internship should be repeated according to the Peyton scheme.

**Do not** hand over the form to the student(s) before mini-CEX.

The completed sheet will be handed in by the student to the senior physician for discussion and will remain in the radiology department.

**Mini-Clinical Evaluation Exercise - Education/ Preparation: CT/ MRI**

**General-RAD Paediatric-RAD Neuro-RAD**

**Name: ______________________**

**Semester: ______________________**

**Date: ______________________**

| **Competence** | Worthy of improvement | Requirements met | Excellent performance |
| --- | --- | --- | --- |
| Checking patient data |  |  |  |
| Evaluation of laboratory values (Crea, GFR, TSH) and medication (e.g. metformin). If necessary, explanation of preparatory measures (e.g. pre-watering) |  |  |  |
| Full completion of questionnaire (document important information) |  |  |  |
| Asking for contraindications (e.g. allergies, metallic implants in MRI) |  |  |  |
| Indication of risks (e.g. allergic reaction, extravasation, possible loudness / contrast medium deposits / NSF / deterioration of renal function / thyrotoxic crisis / radiation exposure) |  |  |  |
| Describing of the procedure and explaining special preparation measures (e.g. depositing metallic objects before an MRI) |  |  |  |
| Inform patient about any uncertainties and ask for possible questions, answer them if necessary. |  |  |  |

**Today the student has the requirements for the above-mentioned skills...**

... not fulfilled (= **significant deficiencies** regarding a technically correct, complete, and flowing course)

... fulfilled (= **at least sufficiently technically correct**, complete and fluent to pass)

... excellently fulfilled (= excellent, **far above-average** performance)

Optional feedback to the student(s):

Strengths:

Suggestions for improvement:

Name of the examining physician:

Signature:

**Implementation of the Mini-CEX**

In order to optimize the learning outcome, the educational interview (CT/MRI) should be taught to the students in the following fixed Peyton scheme:

| 1. Demonstration | The assistant doctor performs an explanatory talk at normal/routine speed. The student observes. |
| --- | --- |
| 2. Deconstruction | The assistant doctor gives a detailed consultation and explains. The student may ask if necessary. |
| 1. Understanding | According to the student's information, the assistant doctor conducts an educational interview. If necessary, these will be corrected and supplemented. |
| 1. Performance | The student conducts an independent information discussion under supervision. |

The independent education of a patient is then evaluated by the assistant physician with the mini-CEX sheet. Incomplete or insufficient competencies are then discussed. If a debriefing is necessary for a large number of competences, i.e. if there are considerable deficiencies, the section of the daily internship should be repeated according to the Peyton scheme.
